# Public Spirit and Politics

**Published:** 1900-10

**Authors:** 


### PUBLIC SPIRIT AND POLITICS.

AFTER many weary days of waiting and discomfort; after many nights of almost unbearable stench, befouling the city, and dooming it in the eyes of casual visitors, the Board of Aldermen passed at the first meeting in September, an ordinance regulating the drying of grain. This is just one more illustration of the type of unmerciful administration under which Buffalo is at present struggling. Not that Buffalo is any worse off regarding its Board of Aldermen than any other city, for boards of aldermen are much alike all over these wide and expansive United States; but it is worse off as regards the local pride exhibited by its city fathers. Not even Chicago, notorious for its corruption, not even New York, infamous for its public waste, would permit the curse of the stench of drying rotten grain which has besmirched Buffalo. Chicago and New York politicians are always on the lookout for the main chance, but they have a certain amount of local pride. They may plunder capitalists and corporations and taxpayers with dispassionate equanimity, but they never overlook local pride, and they have that element of horse-sense which, while it may admit of dirty streets, will not tolerate a stink emanating from every section of the town.

Travelers formed a mighty poor opinion of Buffalo while the grain drying nuisance was in evidence, and left the city believing the odor which assailed their nostrils was an every-day affair. The Board of Aldermen while at fault in the matter for not promptly prohibiting the drying of grain within the city limits, is not alone to blame. It has often been said that some men will do anything for money. That is true in this case at any rate. It would appear to the casual observer that no job would be too foul for the business men of Buffalo who engaged in the highly delectable pursuit of drying rotten grain for a few dollars. Every malt house in Buffalo, with two exceptions, dried the grain and added to the general nausea which the fumes provoked.

Public opinion was wrathful over the outrage, but what good did it do? The newspapers voicing the indignation of their readers protested and threatened. What good did it do? Dr. Wende went to the Board of Aldermen. What good did it do? And, finally, the Board of Aldermen passed an ordinance controlling the drying of damaged grain. What good did it do? What good did anything do? None at all. The maltsters ignored the newspapers, the Aldermen ignored Dr. Wende, and people were made sick. And now, after the drying is completed, the ordinance becomes of force.

Nevertheless, a maltster informed us that there was no money in drying grain. Maybe not; but the grain is dry. The sun has shone, the hay is made, and Buffalo, as usual, got the worst of it all.

